# Serum Albumin Level as a Predictor of Failure to Rescue in Patients Undergoing Surgery for Spinal Metastases

**DOI:** 10.3390/cancers17213477

**Published:** 2025-10-29

**Authors:** Esli Nájera Samaniego, Rose Fluss, Ali Haider Bangash, Sertac Kirnaz, Saikiran Murthy, Yaroslav Gelfand, Reza Yassari, Rafael De La Garza Ramos

**Affiliations:** 1 Spine Tumor Mechanics and Outcomes Research (TUMOR) Lab, Montefiore Medical Center/Albert Einstein College of Medicine, Bronx, NY 10467, USA; a01377851@tec.mx (E.N.S.);; 2 School of Medicine and Health Sciences, Tecnologico de Monterrey, Mexico City 14380, Mexico; 3 Department of Neurological Surgery, Montefiore Medical Center/Albert Einstein College of Medicine, Bronx, NY 10467, USA

**Keywords:** failure to rescue, serum albumin, hypoalbuminemia, spine surgery, spinal metastases

## Abstract

**Simple Summary:**

Surgical intervention for spinal metastases can provide meaningful benefits in terms of pain reduction, neurologic preservation, and functional maintenance. However, this population is medically vulnerable, and major postoperative complications can rapidly lead to early mortality. This phenomenon, known as failure to rescue, refers to death following a major complication within 30 days of surgery. In this study, we evaluated whether preoperative serum albumin, a biomarker reflecting nutritional/inflammatory status and physiologic reserve, is associated with failure to rescue risk. We found that even modest hypoalbuminemia was independently associated with this outcome, suggesting that albumin may serve as a useful risk stratification marker to support surgical decision-making and optimize preoperative patient management.

**Abstract:**

**Background/Objectives**: Failure to rescue (FTR), defined as the occurrence of a major complication plus death within 30 days, is a key measure of surgical safety. Hypoalbuminemia is a known risk factor for poor outcome in metastatic spinal tumor surgery, yet its association with FTR has not been explored. The purpose of this study is to evaluate serum albumin level as predictor of FTR after surgery for spinal metastases. **Methods**: A total of 1749 patients with disseminated cancer who underwent oncologic surgery for spinal metastases (identified by CPT codes) and met our inclusion criteria were identified in the ACS-NSQIP database (2018–2023). The primary endpoint was FTR, defined as a major complication plus death occurring within 30 days of surgery. Serum albumin was analyzed both as a continuous and categorical variable (hypoalbuminemia < 3.5 g/dL, normal albumin > 3.5 g/dL). Univariable and multivariable logistic regression was performed, adjusting for demographic and operative variables. **Results**: The mean preoperative serum albumin level was 3.63 g/dL (standard deviation = 0.642) and the FTR rate was 4% (71 of 1749). After adjusting for potential confounders such as modified Frailty Index 5, ASA class, functional status, emergent case, and reoperation, higher preoperative albumin levels (OR 0.39 [95% CI 0.26–0.61]; *p* < 0.001) were independently associated with decreased odds of FTR. **Conclusions**: The findings of this study suggest an association between preoperative serum albumin level and FTR in oncologic surgery for spinal metastases. This highlights the importance of albumin assessment for perioperative prognosis, but the findings require further validation.

## 1. Introduction

Over the past two decades, extensive research has demonstrated the benefits of surgical intervention for metastatic spinal disease [1,2]. Surgery in this setting has been associated with improved mobility, greater functional independence, better pain control, enhanced cognitive function, and potentially prolonged survival [2,3]. However, despite these advantages, spinal surgery for metastatic disease carries a substantial risk of perioperative morbidity. Reported complication rates range widely, with short-term mortality estimated to be between 10% and 15% [1]. A systematic review by the European Spine Society reported a major complication rate of 21.9% [3], while another study analyzing data from the American College of Surgeons National Surgical Quality Improvement Program (ACS-NSQIP) reported that 18% of patients experienced major complications [4]. Given these risks, the occurrence of postoperative complications can significantly diminish the expected benefits of surgery, potentially accelerating functional decline or leading to premature mortality [5,6].

A critical metric for evaluating surgical safety is failure to rescue (FTR), the occurrence of a major complication and death within 30 days of surgery [7,8,9]. FTR has emerged as a key performance indicator and a target for quality improvement initiatives. While efforts to reduce FTR have traditionally focused on hospital-specific factors, there remains a gap in understanding the role of patient-specific or operative characteristics in predicting FTR [6,10,11]. Identifying potentially modifiable factors associated with FTR may provide actionable strategies to improve patient outcomes and optimize perioperative care.

Among potential risk factors, preoperative albumin level has gained attention as a determinant of surgical outcomes including postoperative complications [12]. Hypoalbuminemia has been linked to heightened risks of postoperative morbidity and mortality across various procedures and a dose-dependent effect on surgical risk has been suggested [13,14,15].

While prior studies have established a correlation between albumin levels and mortality [12,13,15], the association between albumin and FTR specifically has not been investigated. By evaluating albumin as both a continuous and categorical variable, we aim to determine the extent of this association. Our findings may contribute to the development of more precise risk stratification models, ultimately guiding perioperative optimization strategies to improve patient survival in this high-risk population.

## 2. Materials and Methods

This study was classified as exempt from institutional review board oversight (Protocol No. 2016-6862). It involved a retrospective analysis of publicly available database from the American College of Surgeons National Surgical Quality Improvement Program (ACS-NSQIP) for the years 2018–2023.

Inclusion criteria were (1) a diagnosis of “disseminated cancer” as recorded in the ACS-NSQIP; (2) patients who underwent surgery for metastatic spinal tumors, identified via CPT codes; and (3) complete data on preoperative albumin levels. From the original NSQIP database 136,345 patients had a diagnosis of disseminated cancer, while only 2769 patients were managed with metastatic spinal tumor surgery, 17 patients were excluded due to ventilator dependence, and 525 were excluded for missing preoperative albumin data, as well as 478 patients due to missing case type data, resulting in a final sample of 1749 patients.

Patient-level variables included age at surgery, sex, dependent functional status (classified as partially or totally dependent in activities of daily living), body mass index (BMI), American Society of Anesthesiologists (ASA) classification, modified Frailty Index-5 (mFI-5), chronic steroid use, and preoperative albumin level (g/dL). Surgical variables collected included operative time (in hours), perioperative blood transfusion (intraoperative or within 72 h postoperatively), case type (elective or emergent), number of corpectomies, and whether a fusion procedure was performed (compared to decompression alone).

The primary endpoint was FTR. This was defined as the occurrence of a major complication plus death within 30 days of surgery. Major complications were defined as any of the following: unplanned reoperation, deep surgical site infection, organ space surgical site infection, unplanned reintubation, prolonged ventilation ≥ 48 h, pulmonary embolism, cerebrovascular accident, renal failure, myocardial infarction, cardiac arrest, sepsis, septic shock, or pneumonia. These parameters are all part of the ACS-NSQIP outcome variables.

An exploratory data analysis was conducted initially. Descriptive statistics were used to characterize the study cohort. Univariable logistic regression was performed for each variable, with preoperative serum albumin as the primary independent variable. Albumin was analyzed both as a continuous measure and as a categorical variable (Hypoalbuminemia < 3.5 g/dL, Normal albumin > 3.5 g/dL).

Variables achieving statistical significance in univariable analyses were included in the multivariable logistic regression. However, these covariates were included for its known or plausible association with FTR. Perioperative transfusion reflects intraoperative physiological stress and hemodynamic instability; mFI-5 and dependent functional status capture baseline frailty and functional reserve and emergent surgery represents an acute, high-risk clinical scenario often linked with increased postoperative mortality [16,17]. This combination of covariates provides comprehensive adjustment for patient condition, surgical complexity, and physiological vulnerability. Odds ratios (OR) with 95% confidence intervals (CI) were reported. Multicollinearity was assessed using variance inflation factors (VIFs). Statistical significance was defined as a *p*-value < 0.05. Analyses were performed using STATA software, version 19.5 (StataCorp LLC, College Station, TX, USA).

## 3. Results

**Figure 1:**
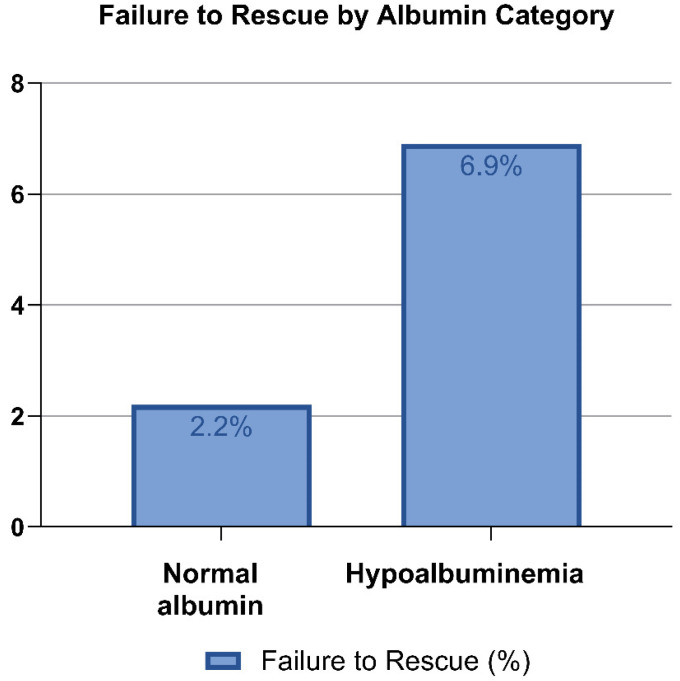
Failure to rescue percentage by albumin level.

A total of 1749 patients who underwent surgery for spinal metastases were included in the analysis; baseline characteristics are described in Table 1. The mean preoperative serum albumin was 3.63 g/dL (SD = 0.642) and 40% had hypoalbuminemia, while 60% had normal albumin levels. Failure to rescue occurred in 71 of 1749 patients (4.1%), the most common complications leading to death were cardiopulmonary and infectious events. Among patients with normal albumin, 23 of 1050 (2.2%) experienced FTR, compared to 48 of 699 (6.9%) in those with hypoalbuminemia as illustrated on Figure 1. Operative characteristics such as median operative time and type of procedure are reported in Table 2.

### 3.1. Albumin Level as Continuous Variable

On univariable analysis, lower preoperative albumin levels were associated higher odds of failure to rescue. Each 1 g/dL increase in albumin was associated with a 66% reduction in the odds of FTR (OR 0.34, [95% CI 0.24–0.48]; *p* < 0.001), indicating that higher albumin is strongly protective. Other variables associated with FTR in univariable analysis included dependent functional status (OR 2.62), higher ASA class (OR 2.13), increasing mFI-5 score (OR 1.59), perioperative transfusion (OR 2.02), and emergent surgery (OR 2.81), as shown in Table 3.

After adjusting for confounders, preoperative albumin level remained an independent predictor of FTR (OR 0.39, [95% CI 0.26–0.61]; *p* < 0.001). Emergent case status (OR 2.40) also remained independently associated with increased odds of FTR, whereas other variables lost statistical significance, as seen in Table 4.

### 3.2. Albumin Level as Categorical Variable

There was a stepwise increase in FTR rates for patients with normal and hypoalbuminemia as shown in Figure 1. These differences were significant on chi-squared testing χ^2^ (1) = 25.91 *p* < 0.001. When analyzing albumin as a categorical variable also in a multivariable model, hypoalbuminemia had nearly a threefold increase in the odds of FTR (OR 2.99, [95% CI 1.67–5.33]; *p* < 0.001) compared to normal albumin level (reference group). Perioperative transfusion (OR = 1.82) and emergent case (OR = 2.52) also remained associated with FTR (Table 5). Variance Inflation Factors (VIFs) were all < 1.2, suggesting no concern for multicollinearity.

To evaluate whether the association between hypoalbuminemia and FTR was influenced by case acuity, a sensitivity analysis restricted to elective cases (*n* = 1041) was performed. Within this subgroup, hypoalbuminemia (<3.5 g/dL) remained associated with higher odds of FTR (OR 2.50, [95% CI 1.12–5.56]; *p* = 0.025), indicating that the relationship persists even among lower-acuity surgical cases.

### 3.3. Selection Bias

To address potential selection bias from excluding patients with missing case type data (*n* = 475), we compared baseline and operative characteristics between included and excluded cohorts (Supplementary Table S1). The excluded cohort demonstrated modestly lower preoperative albumin levels (3.63 g/dL vs. 3.54 g/dL, *p* < 0.001), higher transfusion rates (36.0% vs. 19.5%, *p* < 0.001), and a greater frequency of corpectomy procedures (27.1% vs. 22.5%, *p* = 0.036). These findings suggest that patients with incomplete case-type data were likely higher-acuity cases. Therefore, the direction of bias would be expected to attenuate, rather than exaggerate, the observed association between hypoalbuminemia and FTR.

## 4. Discussion

These findings suggest that lower preoperative serum albumin levels are independently associated with FTR in patients undergoing surgery for metastatic spinal disease. Specifically, every 1 g/dL increase in albumin was associated with 66% reduction in FTR. Failure to rescue reflects the hospital and patient combined ability to survive a major postoperative complication, emphasizing the processes of recognition, escalation, and physiologic resilience rather than complication incidence alone [7,8,18]. In such context, these findings reinforce the value of albumin as a predictive biomarker in this complex patient population, and its potential use for preoperative risk stratification. Beyond being a marker of nutritional status, albumin integrates the inflammatory, metabolic, and systemic reserve, serving as a proxy for physiologic resilience [15,19].

The study aligns with prior NSQIP analyses [1,4,6], but extends the evidence by demonstrating that the association persists even when focusing on FTR, and even among elective cases, where perioperative factors are typically more controlled. This underscores that preoperative albumin is not only a marker of risk for complications, but also of the capacity to recover once complications occur [19,20].

Gelfand et al. reported that severe hypoalbuminemia confers a markedly increased risk of 30-day mortality in metastatic spine surgery patients, emphasizing that even mild reductions in albumin can approach clinical significance [15]. Similarly, Ryvlin et al. highlighted that while a threshold of 3.5 g/dL is commonly used to define hypoalbuminemia, albumin exerts a level-dependent effect on outcomes—with lower levels being consistently associated with worse survival and complications for patients undergoing metastatic spinal tumor surgery [21]. These studies, alongside our findings, underscore the critical importance of routine preoperative albumin assessment and support the concept of a dose–response relationship rather than a fixed cutoff.

Given that hypoalbuminemia may result from a combination of malnutrition and inflammatory processes, our work is consistent with the literature suggesting that albumin cutoff values might be overly simplistic [21,22]. A revised threshold or a more nuanced, continuum-based approach, integrating albumin with other inflammatory markers (e.g., CRP, NLR), may better capture the underlying inflammatory burden and improve risk stratification [14,19]. However, it is important to emphasize that albumin itself is a biologic marker of systemic inflammation and capillary permeability, not a directly modifiable substrate.

Hypoalbuminemia reflects the redistribution of albumin into the interstitium, increased degradation, and shortened half-life secondary to inflammatory signaling, rather than a simple deficit amenable to replacement [19,23]. Consequently, while albumin infusion has not been shown to improve outcomes, holistic preoperative optimization based on nutritional support, inflammation control, and prehabilitation, may enhance both baseline physiologic reserve and postoperative rescue potential, potentially reducing perioperative complications and mortality [24,25,26]. Which is particularly relevant in oncology patients who have undergone chemotherapy, radiotherapy, or prior surgeries, where chronic inflammation and catabolism further deplete systemic resilience

Its validity as a clinically meaningful endpoint is reinforced by data from oncologic surgery [6,9,27]. In colorectal cancer resections, a decade-long analysis demonstrated that improvements in FTR explained most of the observed reduction in mortality, despite stable complication rates, underscoring the impact of effective rescue strategies on patient survival [28,29]. Similarly, in lung cancer surgery, between hospital variation in FTR rather than complication incidence itself was more strongly associated with operative mortality, highlighting FTR as a key determinant of outcome [30,31].

Paralleling these findings in spine surgery cohorts, Rumalla et al. reported a 7.6% FTR rate among 3,632 patients undergoing cervical decompression and fusion, with frailty (Risk Analysis Index–Revised) and perioperative variables predicting FTR with a C-statistic of 0.901 [6]. In 15,749 thoracolumbar fusion cases, Roy et al. found that higher frailty tiers conferred up to a 45.8-fold increase in FTR odds, and their model achieved a C-statistic of 0.92 [10]. The association between albumin and FTR in the context of spine surgery for metastasis, however, had never been investigated.

In the context of spinal metastasis surgery, where malnutrition, systemic illness, and poor functional status are prevalent, FTR offers a pragmatic, patient-centered endpoint that captures both complication occurrence and the individual’s capacity to recover [32,33]. By integrating albumin into this framework, our study highlights the need for individualized, risk-adjusted metrics that incorporate both biologic and system-level factors to better reflect real-world outcomes.

Several limitations inherent to our study must be acknowledged. First, the retrospective design precludes definitive conclusions about causality between hypoalbuminemia and FTR. Despite multivariable adjustments, unmeasured confounding factors, such as the degree of systemic inflammation, tumor burden, and specific nutritional interventions, could influence the observed associations. Additionally, our reliance on a single preoperative albumin measurement does not account for dynamic changes in nutritional and inflammatory status over the perioperative period. The lack of standardized albumin cutoff values across studies, as highlighted by previous studies, further complicates the interpretation of our findings [21,22]. Similarly, our study does not account for variability in laboratory techniques, regional practices, or other factors that may limit direct extrapolation.

Additionally, patients excluded for missing case type data exhibited lower preoperative albumin levels and higher transfusion rates, indicating that incomplete documentation was more common among higher-acuity cases. This pattern introduces the possibility of selection bias, as patients with more comprehensive preoperative evaluations, and therefore lower physiologic risk, were more likely to be included in the analytic cohort. Consequently, our findings may underestimate the true association between hypoalbuminemia and FTR. This limitation also affects generalizability, as the analytic cohort likely reflects centers and circumstances with more complete data capture and systematic preoperative testing. In real-world settings, where data collection and preoperative optimization are more variable, the magnitude of risk associated with low albumin may be greater. Future studies should incorporate standardized preoperative data capture or apply multiple imputation methods to address missingness and prospectively validate whether adjusting for these factors alters the association between albumin and postoperative FTR.

Lastly, although our study focused on FTR as a key outcome, other important endpoints such as long-term functional recovery, quality of life, and detailed complication profiles were not fully explored.

## 5. Conclusions

Our findings highlight the critical role of preoperative serum albumin as a predictor of FTR in patients with metastatic spinal disease. Interestingly, each 1 g/dL increase in albumin was associated with a 66% reduction in the odds of failure to rescue. This underscores the necessity of further research into patient optimization and suggests that current albumin thresholds may need re-evaluation. The converging evidence from our study and the literature calls for well-designed studies to directly assess whether the standard cutoff values are sufficient for risk stratification in this patient population, given the significant differences in outcomes. On the other hand, prospective studies are needed for targeted interventions that improve outcomes in patients undergoing surgery for metastatic spinal disease. Future research should also aim to integrate serum albumin into predictive models alongside other markers of inflammation, nutritional status, and frailty to enhance decision-making in this vulnerable patient group. Collectively, these efforts could contribute to enhanced risk stratification and improved perioperative care.

## Figures and Tables

**Table 1:** Baseline characteristics of 1749 patients.

Parameter	Value, %
Age (mean, SD)	62, 12
Male sex (%)	1044 (59.6%)
Dependent functional status (%)	158 (9.06%)
BMI (mean kg/m^2^, SD)	27.3, 6.2
ASA class (median, IQR)	3, 3–3
Modified Frailty Index 5 (continuous)	
0	806 (46.1%)
1	616 (35.2%)
2	266 (15.1%)
3	61 (3.4%)
Chronic steroid use (%)	358 (20.4%)
Mean preoperative albumin level (g/dL, SD)	3.63, 0.642
Normal albumin (%)	1050 (60%)
Hypoalbuminemia (%)	699 (40%)
Elective case	1041 (59.5%)
Emergent case	708 (40.5%)

SD—standard deviation; BMI—body mass index.

**Table 2:** Operative characteristics of 1749 patients.

Parameter	Value, %
Operative time (median hours, IQR)	3.1 (2.1–4.6)
Perioperative transfusion (%)	297 (16.98%)
Corpectomy (%)	395 (22.55%)
Fusion procedure (%)	990 (56.5%)

**Table 3:** Univariable logistic regression of factors associated with failure to rescue.

Parameter	Odds Ratio	95% Confidence Interval	*p*-Value
Age (continuous)	1.01	0.99–1.03	0.264
Male sex	1.53	0.91–2.55	0.103
Dependent functional status	2.62	1.68–4.08	<0.001 *
BMI (continuous)	0.97	0.93–1.01	0.198
ASA class	2.13	1.43–3.18	<0.001 *
Modified Frailty Index 5 (continuous)	1.59	1.24–2.05	<0.001 *
Chronic steroid use	1.55	0.91–2.63	0.104
Preoperative albumin level (continuous)	0.34	0.24–0.48	<0.001 *
Hypoalbuminemia (<3.5)	3.88	2.36–6.39	<0.001 *
Operative time (continuous)	0.99	0.99–1.00	0.417
Perioperative transfusion	2.02	1.13–3.68	0.016 *
Corpectomy	0.76	0.41–1.40	0.385
Fusion procedure	0.73	0.45–1.18	0.212
Emergent case	2.81	1.71–4.63	<0.001 *

* Included in the multivariable regression; BMI: body mass index.

**Table 4:** Multivariable logistic regression of factors associated with failure to rescue (albumin as continuous variable).

Parameter	Odds Ratio	95% Confidence Interval	*p*-Value
Perioperative transfusion	1.81	0.99–3.30	0.050
Modified Frailty Index 5 (continuous)	1.12	0.79–1.59	0.521
Dependent functional status	1.53	0.82–2.87	0.176
Preoperative albumin level (continuous)	0.39	0.26–0.61	<0.001 *
Emergent case	2.40	1.36–4.33	<0.003 *

* Statistically significant result.

**Table 5:** Multivariable logistic regression of factors associated with failure to rescue (albumin as categorical variable).

Parameter	Odds Ratio	95% Confidence Interval	*p*-Value
Perioperative transfusion	1.82	1.00–3.31	0.047 *
Modified Frailty Index 5 (continuous)	1.13	0.80–1.60	0.478
Dependent functional status	1.63	0.88–3.02	0.119
Hypoalbuminemia (<3.5)	2.99	1.67–5.33	<0.001 *
Emergent case	2.52	1.41–4.49	0.002 *

* Statistically significant result.

## Data Availability

Data is available for review upon reasonable request.

## Supplementary Materials

The following supporting information can be downloaded at: https://www.mdpi.com/article/10.3390/cancers17213477/s1, Supplementary Table S1. Comparison between case type and missing case type

## Abbreviations

The following abbreviations are used in this manuscript:

ACS-NSQIP	American College of Surgeons National Surgical Quality Improvement Program
FTR	Failure to Rescue
BMI	Body Mass Index
ASA	American Society of Anesthesiologists
mFI-5	modified Frailty Index-5

## References

[1] Schoenfeld A.J., Le H.V., Marjoua Y., Leonard D.A., Belmont P.J., Bono C.M., Harris M.B. (2016). Assessing the Utility of a Clinical Prediction Score Regarding 30-Day Morbidity and Mortality Following Metastatic Spinal Surgery: The New England Spinal Metastasis Score (NESMS). Spine J..

[2] Ando K., Kobayashi K., Machino M., Ota K., Morozumi M., Tanaka S., Imai R., Nishida Y., Ishiguro N., Imagama S. (2019). Fusion Surgery with Instrumentation Following Carbon Ion Radiotherapy for Primary Lumbar Tumors: A Case Series. J. Clin. Neurosci..

[3] Tarawneh A.M., Pasku D., Quraishi N.A. (2021). Surgical Complications and Re-Operation Rates in Spinal Metastases Surgery: A Systematic Review. Eur. Spine J..

[4] Boaro A., Wells M., Chi J., Lu Y., Smith T.R., Groff M.W., Zaidi H. (2020). A National Surgical Quality Improvement Program Analysis of Postoperative Major and Minor Complications in Patients with Spinal Metastatic Disease. World Neurosurg..

[5] Paulino Pereira N.R., Ogink P.T., Groot O.Q., Ferrone M.L., Hornicek F.J., van Dijk C.N., Bramer J.a.M., Schwab J.H. (2019). Complications and Reoperations after Surgery for 647 Patients with Spine Metastatic Disease. Spine J..

[6] Rumalla K.C., Covell M.M., Skandalakis G.P., Rumalla K., Kassicieh A.J., Roy J.M., Kazim S.F., Segura A., Bowers C.A. (2024). The Frailty-Driven Predictive Model for Failure to Rescue among Patients Who Experienced a Major Complication Following Cervical Decompression and Fusion: An ACS-NSQIP Analysis of 3632 Cases (2011–2020). Spine J..

[7] Gleeson E.M., Clarke J.R., Morano W.F., Shaikh M.F., Bowne W.B., Pitt H.A. (2019). Patient-Specific Predictors of Failure to Rescue after Pancreaticoduodenectomy. HPB.

[8] Portuondo J.I., Shah S.R., Singh H., Massarweh N.N. (2019). Failure to Rescue as a Surgical Quality Indicator: Current Concepts and Future Directions for Improving Surgical Outcomes. Anesthesiology.

[9] Lafonte M., Cai J., Lissauer M.E. (2019). Failure to Rescue in the Surgical Patient: A Review. Curr. Opin. Crit. Care.

[10] Roy J.M., Segura A.C., Rumalla K., Skandalakis G.P., Covell M.M., Bowers C.A. (2023). A Predictive Model of Failure to Rescue After Thoracolumbar Fusion. Neurospine.

[11] Kang T., Park S.Y., Lee J.S., Lee S.H., Park J.H., Suh S.W. (2020). Predicting Postoperative Complications in Patients Undergoing Lumbar Spinal Fusion by Using the Modified Five-Item Frailty Index and Nutritional Status. Bone Jt. J..

[12] Hussain A.K., Cheung Z.B., Vig K.S., Phan K., Lima M.C., Kim J.S., Di Capua J., Kaji D.A., Arvind V., Cho S.K. (2019). Hypoalbuminemia as an Independent Risk Factor for Perioperative Complications Following Surgical Decompression of Spinal Metastases. Glob. Spine J..

[13] He Z., Zhou K., Tang K., Quan Z., Liu S., Su B. (2020). Perioperative Hypoalbuminemia Is a Risk Factor for Wound Complications Following Posterior Lumbar Interbody Fusion. J. Orthop. Surg. Res..

[14] Chen S.-H., Zhang B.-F., Zhang Y.-M. (2024). The Association between Prealbumin Concentration at Admission and Mortality in Elderly Patients with Hip Fractures: A Cohort Study. Arch. Osteoporos..

[15] Gelfand Y., De la Garza Ramos R., Nakhla J.P., Echt M., Yanamadala V., Yassari R. (2021). Predictive Value of Hypoalbuminemia and Severe Hypoalbuminemia in Oncologic Spine Surgery. Clin. Neurol. Neurosurg..

[16] Piscopo A.J., Park B.J., Perez E.A., Ternes S., Gold C., Carnahan R., Yamaguchi S., Kawasaki H. (2023). Predictors of Survival After Emergent Surgical Decompression for Acutely Presenting Spinal Metastasis. World Neurosurg..

[17] Luksanapruksa P., Buchowski J.M., Hotchkiss W., Tongsai S., Wilartratsami S., Chotivichit A. (2017). Prognostic Factors in Patients with Spinal Metastasis: A Systematic Review and Meta-Analysis. Spine J..

[18] Leeds I.L., Kachalia A., Haut E.R. (2021). Rescuing Failure to Rescue-Patient Safety Indicator 04 on the Brink of Obsolescence. JAMA Surg..

[19] Soeters P.B., Wolfe R.R., Shenkin A. (2019). Hypoalbuminemia: Pathogenesis and Clinical Significance. JPEN J. Parenter. Enter. Nutr..

[20] Camino-Willhuber G., Tani S., Schonnagel L., Caffard T., Haffer H., Chiapparelli E., Sarin M., Shue J., Soffin E.M., Zelenty W.D. (2023). Association of Frailty and Preoperative Hypoalbuminemia with the Risk of Complications, Readmission, and Mortality After Spine Surgery. World Neurosurg..

[21] Ryvlin J., Seneviratne N., Bangash A.H., Goodwin C.R., Weber M.H., Charest-Morin R., Shin J.H., Versteeg A.L., Fourman M.S., Murthy S.G. (2025). The Utilization of Hypoalbuminemia as a Prognostic Metric in Patients with Spinal Metastases: A Scoping Review. Brain Spine.

[22] De la Garza Ramos R., Charest-Morin R., Goodwin C.R., Zuckerman S.L., Laufer I., Dea N., Sahgal A., Rhines L.D., Gokaslan Z.L., Bettegowda C. (2025). Malnutrition in Spine Oncology: Where Are We and What Are We Measuring?. Glob. Spine J..

[23] Abedi F., Zarei B., Elyasi S. (2024). Albumin: A Comprehensive Review and Practical Guideline for Clinical Use. Eur. J. Clin. Pharmacol..

[24] Mazzaferro E.M., Edwards T. (2020). Update on Albumin Therapy in Critical Illness. Vet. Clin. N. Am. Small Anim. Pract..

[25] Soeters P., Bozzetti F., Cynober L., Forbes A., Shenkin A., Sobotka L. (2017). Defining Malnutrition: A Plea to Rethink. Clin. Nutr..

[26] Wiedermann C.J. (2021). Hypoalbuminemia as Surrogate and Culprit of Infections. Int. J. Mol. Sci..

[27] Roy J.M., Rumalla K., Skandalakis G.P., Kazim S.F., Schmidt M.H., Bowers C.A. (2023). Failure to Rescue as a Patient Safety Indicator for Neurosurgical Patients: Are We There yet? A Systematic Review. Neurosurg. Rev..

[28] Christina N.M., Tjahyanto T., Lie J.G., Santoso T.A., Albertus H., Octavianus D., Putri D.A.U.I., Andrew J., Jatinugroho Y.D., Shiady C. (2023). Hypoalbuminemia and Colorectal Cancer Patients: Any Correlation?: A Systematic Review and Meta-Analysis. Medicine.

[29] Wells C.I., Varghese C., Boyle L.J., McGuinness M.J., Keane C., O’Grady G., Gurney J., Koea J., Harmston C., Bissett I.P. (2023). “Failure to Rescue” Following Colorectal Cancer Resection: Variation and Improvements in a National Study of Postoperative Mortality. Ann. Surg..

[30] Farjah F., Backhus L., Cheng A., Englum B., Kim S., Saha-Chaudhuri P., Wood D.E., Mulligan M.S., Varghese T.K. (2015). Failure to Rescue and Pulmonary Resection for Lung Cancer. J. Thorac. Cardiovasc. Surg..

[31] Parkin C.J., Moritz P., Kirkland O., Glover A. (2021). What Is the Accuracy of the ACS-NSQIP Surgical Risk Calculator in Emergency Abdominal Surgery? A Meta-Analysis. J. Surg. Res..

[32] Johnston M.J., Arora S., King D., Bouras G., Almoudaris A.M., Davis R., Darzi A. (2015). A Systematic Review to Identify the Factors That Affect Failure to Rescue and Escalation of Care in Surgery. Surgery.

[33] Silber J.H., Williams S.V., Krakauer H., Schwartz J.S. (1992). Hospital and Patient Characteristics Associated with Death after Surgery. A Study of Adverse Occurrence and Failure to Rescue. Med. Care.

