# Supplementary material for: Serum Albumin Level as a Predictor of Failure to Rescue in Patients Undergoing Surgery for Spinal Metastases
Source: Cancers (Basel). 2025 Oct 29;17(21):3477. doi: 10.3390/cancers17213477 (PMC12609069; doi:10.3390/cancers17213477)
Supplement: Supplementary file 1 [file cancers-17-03477-s001.zip › cancers-3909574-supplementary.pdf]

Table S1. Comparison between case type and missing case type.

Variable	Case Type Recorded (n = 1749)	Missing Case Type (n = 475)	*p*-Value
Age (mean, SD)	62.54	62.80	0.068
Preoperative albumin level (continuous)	3.63	3.54	<0.001
BMI (continuous)	27.33	27.30	0.097
Operative time (continuous)	3.58	3.74	0.121
Male sex (n, %)	1045, 59.6%	285, 60.4%	0.742
ASA class (n, %)			0.641
1–2	160, 9.1%	39, 8.3%	
3	1187, 67.6%	311, 65.9%	
4-6	408, 23.3%	122, 25.8%	
Modified Frailty Index 5			0.557
0	809, 46.1%	203, 43%	
1	618, 35.2%	174, 36.9%	
2	267, 15.2%	74, 15.7%	
3	61, 3.5%	21, 4.5%	
Chronic steroid use	362, 20.6%	79, 16.7%	0.060
Hypoalbuminemia (<3.5)	587, 33.5%	201, 42.6%	<0.001
Perioperative transfusion	299, 19.5%	170, 36%	<0.001
Corpectomy	395, 22.5%	128, 27.1%	0.036
Fusion procedure	991, 56.6%	281, 59.5%	0.232
